# How great white sharks nourish their embryos to a large size: evidence of lipid histotrophy in lamnoid shark reproduction

**DOI:** 10.1242/bio.017939

**Published:** 2016-09-15

**Authors:** Keiichi Sato, Masaru Nakamura, Taketeru Tomita, Minoru Toda, Kei Miyamoto, Ryo Nozu

**Affiliations:** Okinawa Churashima Research Center, Okinawa Churashima Foundation, 888 Motubu, Okinawa Pref. 905-0206, Japan

**Keywords:** White shark, Reproduction, Histotrophy, Oophagy, Uterus

## Abstract

The great white shark (*Carcharodon carcharias*) exhibits viviparous and oophagous reproduction. A 4950 mm total length (TL) gravid female accidentally caught by fishermen in the Okinawa Prefecture, Southern Japan carried six embryos (543-624 mm TL, three in each uterus). Both uteri contained copious amounts of yellowish viscous uterine fluid (over 79.2 litres in the left uterus), nutrient eggs and broken egg cases. The embryos had yolk stomachs that had ruptured, the mean volume of which was approximately 197.9 ml. Embryos had about 20 rows of potentially functional teeth in the upper and lower jaws. Periodic acid Schiff (PAS)-positive substances were observed on the surface and in the cytoplasm of the epithelial cells, and large, secretory, OsO4-oxidized lipid droplets of various sizes were distributed on the surface of the villous string epithelium on the uterine wall. Histological examination of the uterine wall showed it to consist of villi, similar to the trophonemata of Dasyatidae rays, suggesting that the large amount of fluid found in the uterus of the white shark was likely required for embryo nutrition. We conclude that: (1) the lipid-rich fluid is secreted from the uterine epithelium only in early gestation before the onset of oophagy, (2) the embryos probably use the abundant uterine fluid and encased nutrient eggs for nutrition at this stage of their development, and (3) the uterine fluid is the major source of embryonic nutrition before oophagy onset. This is the first record of the lipid histotrophy of reproduction among all shark species.

## Abstract (precis)

**Summary:** Great white sharks (*Carcharodon carcharias*) nourish their embryos with a lipid-rich ‘milk’ secreted by uterine epithelium during the early gestation period, before the onset of oophagy.

## INTRODUCTION

Matrotrophy is common in Chondrichthyes and several modes of embryonic nourishment have been documented, such as histotrophy (either lipid or mucoid), oophagy (adelphophagy), and placentotrophy (Hamlett et al., 2005). In lamniform sharks, oophagy is one of the primary modes of embryonic nutrition (Wourms, 1977); indeed, it is a defining characteristic of the group although there are a few species whose reproductive modes have yet to be observed. Within the lamnoids, *Carcharias taurus*, *Lamna nasus*, and *Isurus oxyrinchus* are relatively well-studied, providing reproductive reference models against which to contrast other lamniform sharks. Gilmore (1993) suggested that the embryos of *C. taurus* pass through at least six nutritive phases during gestation, including: yolk-sac yolk, uterine fluid, egg capsules containing other embryos, and egg cases containing unfertilized ova. The reproduction of the great white shark *C. carcharias* (hereafter referred to as ‘white shark’) was evaluated recently (Castro, 2011) based on several gravid specimens caught in Japan, New Zealand, and some unpublished photographs of embryos uploaded on web-pages. A gravid female recorded from Toyo-cho, Japan (Uchida et al., 1996) contained ten young that measured 135-151 cm, and another from New Zealand (Francis, 1996) contained seven young measuring 143.0 and 144.9 cm TL (only two of them were measured). These embryos did not exhibit any signs of a swollen external yolk stomach and appeared to be near-term. To our knowledge, there are several records of early to mid-term embryos that have been examined and measured by biologists (Christiansen et al., 2014), but little is known about white shark matrotrophy in the early gestation period between the end of yolk-dependency and the onset of oophagy.

Histological examination of the mature uterus is essential to evaluate the type of matrotrophy. Recent studies suggest many lecithotrophic sharks have secretory tissues associated with their uterine walls, implying that maternal nourishment likely occurs in these species (Hamlett et al., 2005; Cotton et al., 2015). These forms are classified as ‘mucoid histotrophy’ (Conrath and Musick, 2012) when mucus-secreting cells are evident in the uterus; ‘embryotrophy’ (Castro et al., 2016) when there is lipid-free but energy-rich uterine fluid evident, or ‘lipid histotrophy’ when trophonemata produce copious amounts of yellowish, milky secretions (currently known only in batoids). No histological or histochemical examination has ever been conducted on a white shark uterus throughout its gestation period because the white shark is listed as ‘Vulnerable’ on the IUCN Red List (http://www.iucnredlist.org, 2016), therefore, there have been very few opportunities to observe pregnant females or to study the histology of the uterus.

Present research examined a gravid female specimen which was incidentally and legally caught by the fishery off Yomitan Village, Okinawa Prefecture, Japan, on 13 February 2014. The white shark is a species with no commercial value in this area, and the landed specimens were donated for scientific research by the Yomitan Fishery Cooperative. This specimen has provided the scientific community with an opportunity to significantly further our knowledge of white shark reproduction. The specimen contained rare embryos at a relatively early stage of gestation in both uteri, with a large amount of yellowish, milky uterine fluid. We describe the morphological features of these embryos and the microstructures of the uterine wall of the mother, below.

## RESULTS

### Characteristics of embryos in uterus

**Fig. 1.**
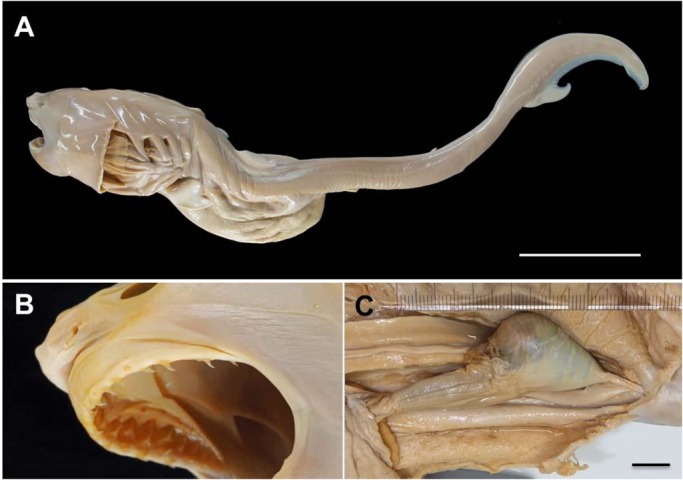
**A white shark embryo obtained from gravid female.** (OCF-P 20140213-7, 543 mm TL, male; scale bar=10 cm). (A) Lateral view of the embryo. (B) The embryonic teeth on upper jaw, and (C) spiral intestine (scale bar=1 cm).

Table 1 shows the size and proportional measurements of the embryos. The total lengths, weights and external features suggested that most of the embryos were at the same developmental stage, though a 543 mm TL embryo (Fig. 1A) was somewhat smaller than the others. The body was entirely a pale whitish colour, without pigmentation or colour pattern. The trunk region behind cloaca was very narrow, while all paired and unpaired fins were small. The caudal fin was not lunate shaped, and its lower lobe was undeveloped. About 20 rows of ‘embryonic teeth’ (*sensu* Gilmore, 1993) were present on both upper and lower jaws (Fig. 1B); they were triangular and ∼2 mm in crown height, and these teeth were half-erect and probably functional. Such embryonic teeth have been examined and documented in other lamniform genera, such as *Isurus*, *Carcharias*, and *Lamna*, and are believed to be used for puncturing the capsule of nutritive eggs (Gilmore, 1993; Francis and Stevens, 2000; Shimada, 2002). Pharyngeal arches were expanded laterally, and the pharyngeal cavity was greatly opened (Fig. 1A). The stomach was distended but ruptured at the postero-ventral portion, probably filled with uterine fluid or yolk substances; its average approximate volume was estimated to be 197.9 ml (s.d.=59.9 ml). The external yolk-sac had already disappeared from the ventral surface of the body, but small rudiments of the internal yolk sac were evident in the body cavity which was connected to the intestine by the ductus vitello-intestinalis. The spiral intestine at posterior portion (Fig. 1B,C) has a hard obstruction with a fine-grained crystalline material which was easily dissolved by acetic acid, which may prevent the embryos excreting into the uterus.

**Table 1.**
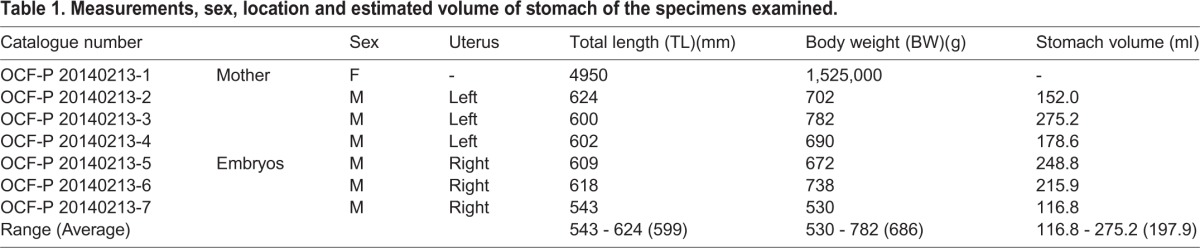
**Measurements, sex, location and estimated volume of stomach of the specimens examined.**

### Uterine environment

**Fig. 2.**
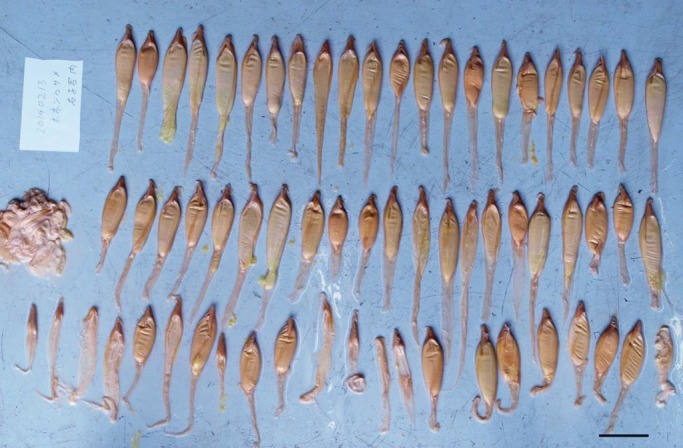
**Nutrient egg capsules.** Sixty encased nutrient eggs and some broken cases found from the right uterus of the mother shark (OCF-P 20140213-1). Scale bar=10 cm.

Each uterus was filled with viscoid, yellowish fluid (Figs S3 and S4) similar to the ‘uterine milk’ of the manta rays. The precise volume of the uterine fluid could not be measured because some fluid had been lost. The measured volume of the residual fluid was 79.2 litres (from the left uterus). The left and right uteri contained three embryos each, and at least 60 and 65 egg cases (Fig. 2, Fig. S4) containing small nutrient eggs were present in both left and right uterus. There was also evidence of several broken egg cases. The mean diameter of the naked nutrient eggs was 7.4 mm, and sometimes found in the uterine fluid, but their origins (egg case or stomach of embryos) were not determined.

A precise analysis of the uterine fluid was not carried out in the present study because of the possibility of contamination of the fluid by embryonic stomach contents, external substances and blood from the mother. However, considering the total volumes of the nutrient eggs and the embryonic stomachs, these contaminations were obviously not the major contents of the uterine fluid.

### Histology and histochemistry

#### Uterus

**Fig. 3.**
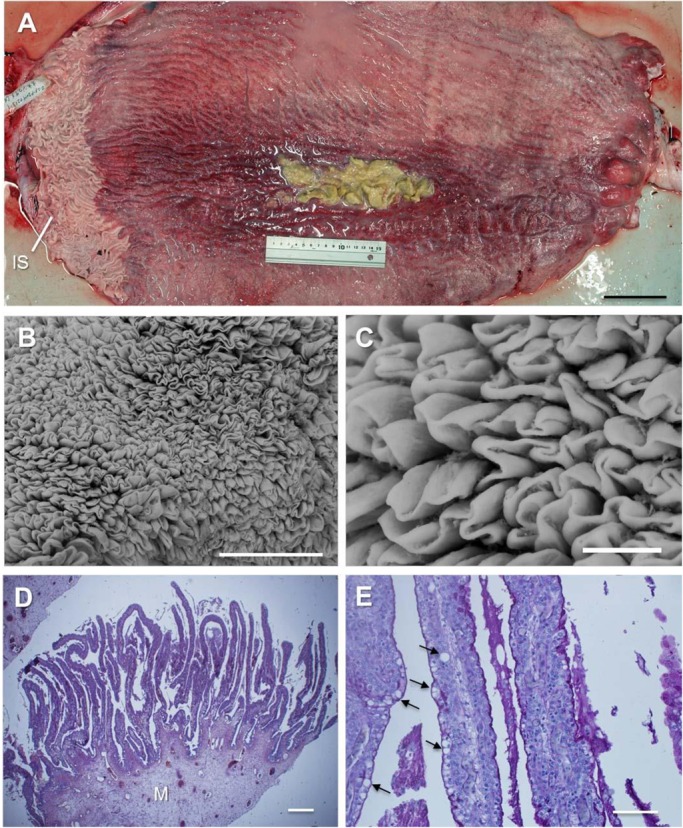
**Structure of the uterus of the white shark *Carcharodon carcharias*.** (A) The left uterus was cut across the dorsal wall. Large, white ridges developed at the isthmus (IS) of the uterus. A linear buttery paste-like substance was seen on the surface of the central part of the uterus. (B,C) Close-up of the surface of the uterus, the lamellae on the side facing the lumen show full development. (D) The lamellae on the epithelial cells showed PAS-staining, the lamellar complexes developed on the thick muscle tissue (M). (E) Strong PAS-staining was observed both in the apical cytoplasm of the epithelial cells on the side facing the lumen and in the substance present in the lamellae. Many vacuoles (black arrows) in various sizes were observed in the apical cytoplasm of epithelial cells. Scale bars=10 cm (A), 1 cm (B), 1 mm (C), 500 µm (D), 250 µm (E).

Observations detailed below are based on the left uterus. A streak of butter-like substance was found on the surface of the central part of the uterus (Fig. 3A). Several large ridges that were white in colour (3.4–7.7 mm in width and 9.3 mm in depth) were observed near the entrance of uterus, merging with the oviducts.

**Fig. 4.**
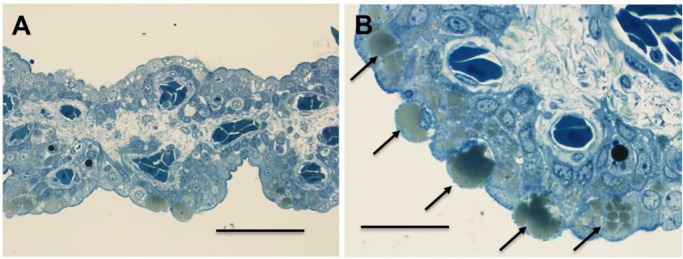
**Low and high magnifications of villus string oxidized by OsO4 (Epon-embedded 1-µm sections).** (A) Blackish lipid droplets (arrows), round in shape and in various sizes were seen in the cytoplasm of some epithelial cells. (B) Some large lipid droplets (arrows) on the surface of epithelial cells, indicating the secretion of lipid droplets into the lumen, are seen. Scale bars=250 µm (A), 50 µm (B).

Observation of the right uterus revealed that the epithelium was abundantly covered with thin lamellar complexes (3.3–4.8 mm in length; Fig. 3B,C). Epithelial cells of the lamellae consisted of two or three layers of cytoplasm-rich cuboidal cells with large, round nuclei. Some epithelial cells facing the lumen showed large vacuoles in their cytoplasm. The central part of the lamellae consisted of fibroblasts and a network of capillaries (Fig. 3E). PAS-positive substances were observed on the surface and cytoplasm of the epithelial cells of the lamellae but not in their large vacuoles and fluid (Fig. 3E). The thick outer wall of the uterus was mainly composed of developed smooth muscle tissues and collagen (Fig. 3D). Many large blackish lipid droplets of different sizes seen in the cytoplasm of some epithelial cells of villous strings (Fig. 4A,B) were oxidized by OsO4. This suggested that many of the vacuoles observed in the epithelial cells were also lipid droplets. Several thick blood vessels were observed in the tissues. Two types of PAS-positive substances were observed in the uterine fluid: PAS-positive granules probably originated from broken nutrient yolk-sac yolk, whereas the PAS-positive fluid was probably secreted from the uterine epithelium.

#### Ovary

**Fig. 5.**
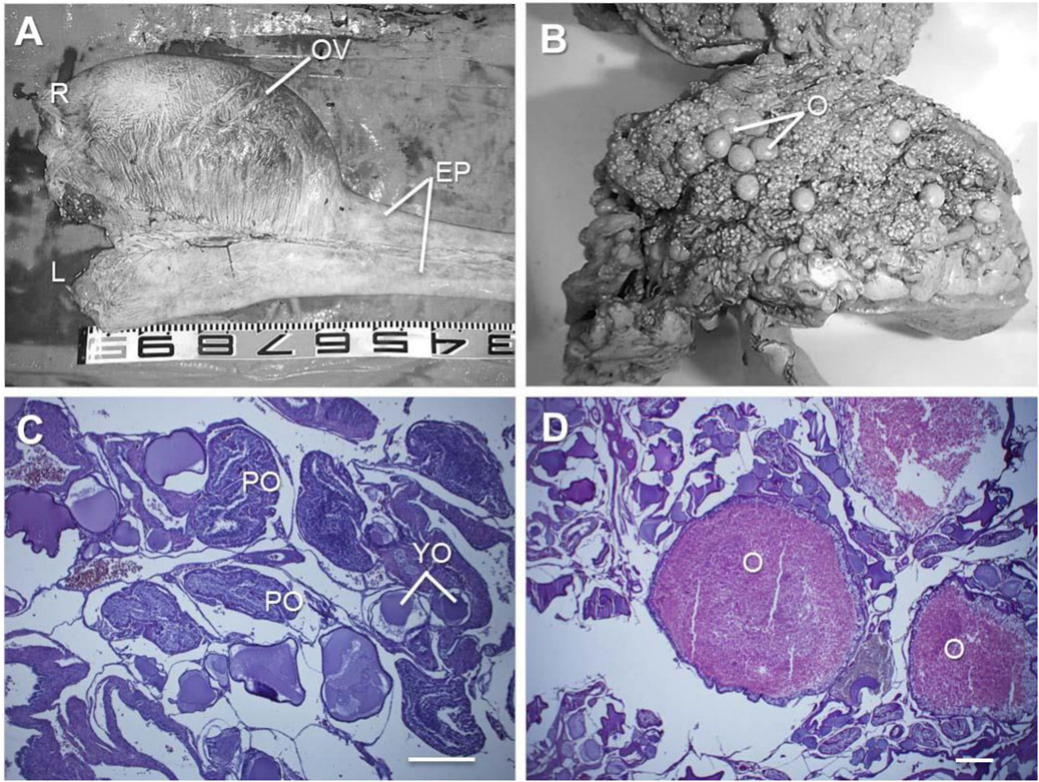
**Ovary of the white shark *Carcharodon carcharies*.** (A) The right ovary (OV) shows full development of the anterior of epigonal organ (EP). (B) A fragment of the ovary after fixation with Bouin's solution. Several yolk-containing oocytes (O) and small oocytes are evident. (C) Histology of ovarian tissue stained with hematoxylin and eosin. Several post-ovulatory follicles (PO) are seen together with young oocytes (YO). (D) Yolk-containing oocytes in the ovary stained with PAS. Yolk granules are strongly stained with PAS. Scale bars=500 μm (C,D).

Only the right ovary was developed and functional, showing a remarkable development of the epigonal organ (Fig. 5A); however, the left ovary was difficult to identify due to its underdevelopment and was covered with a white tissue. Although numerous oval-shaped yolk-containing oocytes (4.5–5.5 mm at the longest diameter) together with small oocytes (less than 1 mm in the longest diameter) were found in the right ovary (Fig. 5B), no well-developed oocytes were longer than 6 mm. Histological observations showed several yolk-containing oocytes, post-ovulatory follicles, and young oocytes before yolk formation within the same ovarian tissue (Fig. 5C). The follicular layers enclosing the yolk-containing oocytes showed hypertrophy or incorporation of the yolk granules into their cytoplasm, indicating oocyte degeneration (Fig. 5D). The yolk granules within the cytoplasm of yolk-containing oocytes were strongly PAS-stained (Fig. 5D).

#### Stomach and intestinal contents of embryos

**Fig. 6.**
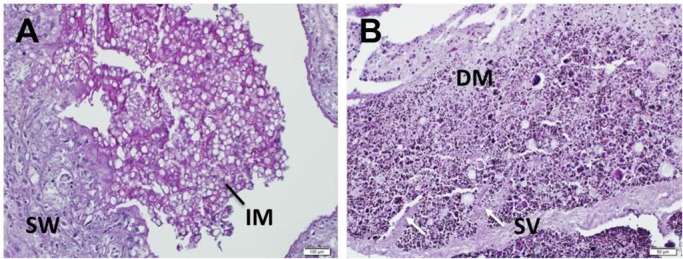
**Cross-section of the stomach wall and spiral intestine of the embryo, stained with PAS.** (A) Surface of the stomach wall (SW) and ingested material (IM) in the minute stomach folds (scale bar=100 μm), and (B) the spiral valves (SV with white arrows) and the digested material (DM) in intestine (scale bar=50 μm).

Histological observation suggested that the epithelial cells of the embryonic stomach do not have a secretory function, instead, the stomach appears to function as storage of nutrients. Embryonic stomach contents contain lipid droplets secreted as vacuoles from the uterine epithelium (Fig. 6A), but no evidence of lipid droplets was found in the spiral intestine of the embryos (Fig. 6B). Numerous PAS-positive digested granules regarded as digests of granules were observed in the spiral intestine.

### Comparative material

**Fig. 7.**
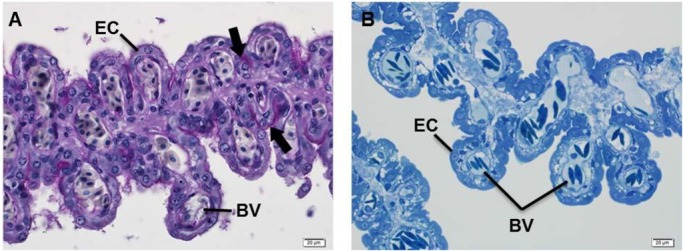
**Histological observation of the left uterus of the comparative specimen in late gestation phase.** (A) Many blood vessels (BV) localized in the uterine lamellae. PAS-positive reactions (arrows) were detected on the surface of the epithelial cells (EC). No lipid droplet was observed in semi-thin section (1 µm) of the uterine lamellae fixed by OsO4 (B). Scale bars=20 µm.

The uteri of the comparative material (OCF-P03062, 5050 mm TL) carried four embryos with full-yolk stomachs in the left uterus only, and contained copious amounts of clear uterine fluid with low viscosity. Histological observations on the left uterus showed that epithelium was composed of a single layer of cells with large and round nuclei (Fig. 7A,B). Many capillary vessels were localized on the inner side of the epithelial cell layer (Fig. 7A,B). PAS-positive reaction was detected only on the outer surface of epithelial cells (Fig. 7A). A semi-thin section of the uterus fixed by OsO4 revealed that cytoplasm of epithelial cells contained no blackish droplet, which suggested that the uterus secreted no lipid droplet (Fig. 7B).

## DISCUSSION

Our morphological and histological examination of early oophagus-stage embryos suggests that a lipid-histotrophic phase likely precedes the oophagus phase. The presence of histotrophic nutrition in lamniform shark reproduction was firstly hypothesized by Gilmore (1983, 1993), based on the study of *Carcharias* *taurus*. Gilmore (1993) suggested that *C. taurus* had at least six phases of embryonic nutrition, and that the fluid secretion from the uterine epithelium was activated around hatching to provide oxygen and nutrition to the embryos in uterus. These conclusions were based on the presence of a highly vascularized uterine epithelium, particularly at the isthmus; however, they did not show any direct histochemical evidence of histotrophy. Gilmore (1983) also documented that numerous trophonemata are present in the uterine wall of *Isurus oxyrinchus* and mentioned that the structure was undoubtedly a major source of respiratory oxygen for the lamnoid sharks.

Our study provides the first direct evidence of uterine secretion of lipid-rich uterine fluid often dubbed ‘uterine milk’ in lamniform sharks. This study indicates that the amount of the uterine fluid was remarkably large, measuring 79.2 litres from the left uterus containing three embryos, and we assume that the fluid is secreted from the uterine epithelium of numerous villi or lamellae. Precise detection of each component of the fluid has not yet been completed, but histochemical staining suggests that the uterine epithelium of villous strings are implicated in the secretion of lipid droplets and at least two types of PAS-positive granular and fluid substances. The lipid secretions in the white shark were highly active and resembled those from the trophonemata of pregnant manta rays (Soma et al., 2013).

It is likely that the white shark embryos in the stage examined in this study consume not only uterine milk but also nutrient eggs. Embryos have functional teeth and 50-70 encased nutrient eggs, and some broken capsules, were found in both left and right uteri. These observations indicate that the embryos may consume some nutrient egg capsules at this stage, but the total numbers of capsules were insufficiently large to exclusively account for the nourishment of three embryos. Hamlett (W.C. Hamlett, PhD thesis, Clemson University, 1983) predicted the level of consumption of egg capsules in *C. taurus* as 1000-1700 during a single pregnancy; if this assumption is applied for present research the egg capsules found in each uterus are much less than the requirements for three embryos. The still developing oocytes (4.5–5.5 mm diameter) and several post-ovulatory follicles (over 6 mm diameter) found in the ovaries indicated a very early stage of yolk supply. These observations suggest that uterine fluid is the major source of nutrition for white shark embryos in early gestation, and that the nutrient egg is the subsequent source in white shark development. It is likely that the importance of the lipid secretion decreases through gestation because ‘uterine milk’ has not been observed in the uterus in late gestation period (Uchida et al., 1996). Our observation on the comparative materials in late gestation phase also showed that the uterus contained clear liquid with low viscosity. In addition, the structure of the uterine epithelium in late gestation was completely different from that in early gestation, and no lipid droplet was seen in the uterine epithelium (Fig. 7A,B). Moreover, the highly vascularized uterine lamellae further increased the contact area to provide oxygen into the uterine fluid. These facts indicate that the uterine epithelium transforms its structure and functions with progress of pregnancy. Likewise, Jensen et al. (2002) observed many pregnant females of *Lamna nasus* in the late gestation period and described the presence of clear intrauterine fluid surrounding the embryos consuming yolk-sac yolk, suggesting that the sharks with oophagus reproduction probably change the composition of uterine fluid over the course of gestation.

This study suggests that white shark reproduction is complex. Embryos probably rely on a changing source of nutrition over the course of their development. The embryos depend on at least three major sources of nutrition, yolk-sac yolk (lecithotrophy) in the initial phase, uterine milk (lipid histotrophy) in the second phase and nutrient eggs (oophagy) in the third phase.

Although present data were obtained from the limited number of specimens, our data are highly suggestive about the strategies that white sharks employ to nourish their embryos to ‘extra-large’ size in the gestation. The combination of maternal input with lipid-rich uterine milk and nutrient yolk may increase the maximum size of full-term embryos *in utero*; however, questions remain regarding whether the other lamniform sharks nourish their embryos in the same way.

## MATERIALS AND METHODS

### Materials examined

Female (six embryos in uteri) [OCF-P 20140213-1 (Fig. S1)], 4950 mm TL, 1525 kg body weight (BW), incidentally caught by coastal set-net off Yomitan, Okinawa Prefecture, 13 February 2014. Embryos: OCF-P 20140213-2, 624 mm TL, 702 g BW, male; OCF-P 20140213-3, 600 mm TL, 782 g BW, male; OCF-P 20140213-4, 602 mm TL, 690 g BW, male; OCF-P 20140213-5, 609 mm TL, 602 g BW, male; OCF-P 20140213-6, 618 mm TL, 738 g BW, male; OCF-P 20140213-7, 543 mm TL, 530 g BW, male. Comparative materials: Female [OCF-P 03062 (Fig. S2)] carrying four embryos (1028–1072 mm TL) in the left uterus, 5050 mm TL, 1476 kg BW, incidentally caught by coastal set-net off Yomitan, Okinawa Prefecture, 1 February 2016. Specimens with catalog numbers have been preserved and kept in the Okinawa Churashima Foundation (OCF). The maternal specimen was dissected and only the head region and skin were retained; tissues were taken for genetic analysis.

### Methods

The individual (OCF-P 20140213-1) was caught as by-catch of a set-net off Yomitan Village, Okinawa Prefecture on 13 February 2014, and samples were collected from the specimen within 24 h.

Proportional measurements of both the mother and embryos were taken as listed in Table 1. Total volume of uterine fluid was measured after dissection, though a considerable amount of the fluid was lost when the specimen was transported in a sling by crane before observation. Embryos were preserved in a 10% formalin solution and two weeks later stored in 80% ethanol solution. Though the expanded stomach of the white shark embryo was originally filled with liquid, the liquid had depleted by the time the embryos were taken from the uterus. Thus, we estimated the approximate volume of the liquid, assuming that the expanded stomach is a simple spheroid shape with its long axis parallel to the body. The equation we used was

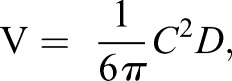
where V (ml) is liquid volume, *C* (cm) is circumference around the stomach at the middle half of the stomach, and *D* (cm) is the antero-posterior depth of the stomach. Circumferences were acquired using a measuring tape; antero-posterior depths were acquired using hand caliper.

For the histological and histochemical observations, tissue samples (approximately 1×1×4 cm) were collected from various parts of the right ovary and left uterus. After overnight fixation with Bouin's solution, these were dehydrated in a series of ethanol concentrations, xylene, and finally mounted in paraffin. The tissues were sectioned into 7-µm-thick sections. Sections for standard histology were stained with hematoxylin and eosin. For the detection of aldehyde and mucosubstances sections were stained with the microscopy Periodic acid-Schiff (PAS) staining kit (Merck, Germany, Cat. No. 1016460001) according to the standard protocol. The epithelial tissues from some parts of the uterus were fixed with Karnovsky's solution (Karnovsky, 1965). After the fixation, tissues were also post-fixed with 1% osmium tetroxide (OsO4) cacodylate buffer. After dehydration, they were embedded in the epoxy resin. One-µm sections were stained with toluidine blue solution to detect lipid secretions in the tissues.
